# How effective are community health workers in managing and preventing perinatal depression in sub-Saharan Africa? A systematic review of quantitative evidence

**DOI:** 10.1093/heapol/czaf084

**Published:** 2025-10-30

**Authors:** Garumma T Feyissa, Enrique R Pouget, Matiwos Soboka, Radyah Ibnat, Tracy Wong

**Affiliations:** Department of Health and Nutrition Sciences, Brooklyn College, City University of New York, 2900 Bedford Avenue, Brooklyn, NY 11210, United States; Department of Health and Nutrition Sciences, Brooklyn College, City University of New York, 2900 Bedford Avenue, Brooklyn, NY 11210, United States; Department of Psychology and Neurosciences, Dalhousie University, 1355 Oxford Street, P.O.BOX 1500, Halifax, NS, Canada B3H 4R2; Department of Health and Nutrition Sciences, Brooklyn College, City University of New York, 2900 Bedford Avenue, Brooklyn, NY 11210, United States; Department of Health and Nutrition Sciences, Brooklyn College, City University of New York, 2900 Bedford Avenue, Brooklyn, NY 11210, United States

**Keywords:** perinatal depression, postnatal depression, community health workers, sub–Saharan Africa, systematic review

## Abstract

The accessibility to the prevention and management of perinatal depression can be improved by using community health workers. This review was aimed at determining the effectiveness of interventions led by community health workers (CHWs) in reducing depressive symptoms and the prevalence of depression during the perinatal period. We conducted a search in PubMed, CINAHL, SCOPUS, and ProQuest Databases of Dissertation and Thesis (PQDT) to locate studies conducted in sub-Saharan Africa. We appraised the quality of eligible studies using standardized critical appraisal instruments from the Joanna Briggs Institute (JBI). We extracted data from the included studies using an a priori prepared data extraction tool. We pooled the findings of the studies using meta-analysis. The initial search yielded 199 studies, out of which we included 16 articles in this review. During the first 3 months after birth, CHW-led preventive psycho-social interventions reduced the risk of depressed mood by 35% [RR = 0.65(0.46,092)] [low-quality evidence]. The interventions reduced the risk of depressed mood by 32% 6-months post-birth [RR = 0.68(0.52, 0.87)] [very low-quality evidence]. The effect of the interventions is sustained through 9–12 months after birth resulting in a reduction in the risk of depressed mood by 38% [RR = 0.72(0.54,0.96)] [low-quality evidence]. Among women with moderate depressive symptoms, compared to usual care, CHW-led therapeutic psycho-social interventions reduced the symptoms by an average of 0.71 [SMD = −0.71 (−0.84, −0.59) units during the first 3 months after birth. The effect lasts 9–12 months after birth [SMD = −0.28 (−0.41, −0.15)] [Moderate-quality evidence]. In conclusion, the work of CHWs may be integrated into the prevention and management of perinatal depression after careful analysis of the feasibility, applicability and meaningfulness of the interventions to local context. High-quality randomized trials may help to inform further optimization of the role of CHWs in reducing the risk of depressed mood and depressive symptoms during perinatal period.

Key messages

- In Sub-Saharan Africa, the role of community health workers (CHWs) in improving accessibility to general primary healthcare has been documented. However, their role in the prevention and management of perinatal depression is unclear.
- This review addressed this gap by assessing how effective CHWs are in the prevention and management of perinatal depression using a systematic review of quantitative studies.
- Low quality evidence supports the use of CWHs-led psychosocial interventions for the prevention of the risk of depressed mood.
- Moderate-quality evidence indicates that psycho-social therapeutic interventions delivered by CHWs lower depressive symptoms among perinatal women with moderate depressive symptoms.
- The current evidence might be utilized with further contextual considerations. Further high-quality trials are required to optimize the role of community health workers in the reduction of incidence of depressed mood and depressive symptoms during perinatal depression.

## Introduction

Worldwide, about 10% of pregnant women and 13% of postnatal women experience a mental health disorder (WHO 2023). Perinatal depression is one of the most common mental health problems during the perinatal period. Perinatal depression is a depressive episode occurring during pregnancy or within the first 6 weeks of postnatal period (according to International Classification of Diseases (ICD-11) definition) (Hong and Zeng 2022 ) or within the first 4 weeks after birth (according to the Diagnostic and Statistical Manual (DSM)-V definition) (Guha 2014). However, the onset of perinatal depression or the symptom duration of perinatal depression may be throughout the first year of the postnatal period or beyond (Radoš et al. 2024).

The prevalence of perinatal depression among women in low- and middle-income countries is estimated to be 25%. More than one-fifth of women in Africa develop depression during each of the antenatal and postnatal periods (Endomba et al. 2021). Perinatal depression is associated with a higher risk of preterm birth, low birth weight, and other adverse birth outcomes (Eastwood et al. 2017 , Dadi et al. 2022 ).

The body of evidence indicates that perinatal depression most often starts during the antenatal period (Wilcox et al. 2021). Hence, early detection and treatment may reduce the severity and the persistence of the symptoms throughout the postnatal period. Global evidence indicates that psycho-social interventions may potentially be used to prevent or treat perinatal depression (Li et al. 2022, Motrico et al. 2023). The World Health Organization (WHO) recommends screening for perinatal depression and anxiety using a validated instrument and diagnostic and management services for women who screen positive. In addition, the WHO recommends psycho-social and/or psychological interventions during the antenatal and postnatal period to treat or prevent perinatal depression and anxiety (WHO 2022).

Globally, psycho-social interventions provided both by non-specialist professionals and lay health workers have been found to be effective in managing perinatal depression (Dennis and Dowswell 2013). Experts recommend the expansion of mental health services through task shifting approach, which also includes assigning the task of delivering mental health services to community health workers (CHWs) (Bolton et al. 2023). Emerging global evidence suggests the potential effectiveness of task sharing approaches for the provision of perinatal mental health services (Prina et al. 2023).

In low- and middle-income countries, including sub-Saharan Africa, CHWs have played substantial roles in the success of other health programs. Some of these contributions were increasing and expanding service delivery, sharing the work burden of health workforce, providing information on prevention and control of different health problems and promotion of health (Ludwick et al. 2020). The contribution of CHWs in the control of communicable diseases, such as human immunodeficiency virus (HIV) and tuberculosis, and in increasing preventive health service coverage such as immunization (Patel and Nowalk 2010) and supplementing the formal health care system has been remarkable (Woldie et al. 2018). By greatly increasing access to essential health services, CHWs have contributed toward the reduction of infant mortalities (Tadesse Zeleke 2022) and maternal mortality (Rieger et al. 2019).

Accordingly, the accessibility of perinatal mental health services, including the prevention and management of perinatal depression, can be improved by using CHWs as agents (Raviola et al. 2019, Connolly et al. 2021). The role of CHWs is especially relevant for the detection, prevention and management of perinatal depression as global evidence is suggesting the potential role of psychological treatment for the management of perinatal depression (Gajaria and Ravindran 2018, Cuijpers et al. 2023). Evidence shows that CHWs can play critical role in managing mild to moderate depressive symptoms (WHO 2022).

Some studies from sub-Saharan Africa have reported that interventions led by community-health workers are effective in reducing depressive symptoms during the perinatal period (Rotheram-Borus et al. 2014a, Kaaya et al. 2022). To generate the best available evidence on the interventions that are feasible, applicable, meaningful and effective in preventing or managing perinatal depression by CHWs, a systematic review of available evidence is required. Evidence on the type and nature of effective interventions that could effectively be delivered by CHWs may inform policy and practice related to the prevention and management of perinatal depression. Through our search in the Cochrane Databases of Review of Effectiveness, PubMed, and CINAHL, we did not find any systematic review that addressed the effectiveness of community health worker interventions to reduce depressive symptoms or the prevalence of depression during the perinatal period in the sub-Saharan African context.

In addition, in the context of many sub-Saharan African countries, through task-sharing approach, CHWs do not just deliver a single type of intervention for a temporary period of time, but are integrated as part of the healthcare system to provide comprehensive and sustainable services (Mupara et al. 2023). While currently available global evidence on psycho-social interventions either lacks CHWs or perinatal depression or is focused on a single intervention type, such as counseling, for the health systems of sub-Saharan African countries, the need for evidence goes beyond that. Because of the need to integrate the activities of CHWs into the healthcare system in a sustainable way, comprehensive evidence is required for the different approaches, types and delivery methods of psycho-social interventions to prevent and manage perinatal depression.

Cognizant of this gap in evidence, this systematic review was aimed to search, appraise and synthesize the best available evidence on the effectiveness of community health worker-led interventions to prevent or treat perinatal depression in sub-Saharan Africa. The synthesized evidence lays groundwork to clarify how sub-Saharan Africa and other low-and-middle income countries use CHWs for task sharing of psycho-social interventions to prevent and manage perinatal depression in the context of shortage of mental health specialists and to increase accessibility of services for perinatal depression at community level.

## Research questions

How effective are interventions led by CHWs in reducing depressive symptoms and the prevalence of depression during the perinatal period in sub-Saharan Africa? Specifically, the review sought to determine

- The effectiveness of interventions led by community health workers in reducing depressive symptoms during the perinatal period,
- The effectiveness of interventions led by community health workers in reducing the prevalence of depression during the perinatal period.

## Methods

This review was conducted according to the Joanna Briggs Institute (JBI) guidance for systematic review of quantitative studies. The report was prepared in accordance with Preferred Reporting Items for Systematic reviews and Meta-analyses (PRISMA) checklist (Page et al. 2021) (Supplementary file S1). We did not register a protocol for this review. We considered the following inclusion criteria for the review.

### Inclusion criteria

#### Population

This review considered perinatal women as a population of interest. Research indicates that perinatal depression often persists throughout the entire postpartum year (Radoš et al. 2024). Hence, for this review, studies of women who received any intervention during pregnancy and/or within 1 year after delivery were considered as eligible, even for those in which the follow-up period for the assessment of the outcomes was open beyond the perinatal period.

#### Intervention

This review considered any psycho-social intervention alone, or combined with other health, nutritional or economic interventions or alone if they were delivered by CHWs. This review adopted the WHO definition of Community Health Workers (World Health Organization 2020), “health workers based in communities (i.e. conducting outreach from their homes and beyond primary health care facilities or based at peripheral health posts that are not staffed by doctors or nurses), who are either paid or volunteer, who are not professionals, and who have fewer than 2 years training but at least some training, if only for a few hours.” This review considered interventions delivered by both salaried and voluntary CHWs.

#### Comparators

This review considered comparators such as, usual interventions, interventions delivered by health professionals or no interventions or alternative modalities/types of interventions delivered by CHWs.

#### Outcome

The primary outcome considered for this review was perinatal depressive symptoms measured by scales, such as Patient Health Questionnaire (PHQ-9), Edinburg Postnatal Depression Scale (EPDS), Hopkins Symptom Checlist-25 (HSCL-25), Hamilton Depression Rating Scale (HDRS), Center for Epidemiologic Studies Depression Scale (CES-D), and other validated measures. As a secondary outcome, we compared the prevalence of depression based on clinical diagnosis or based on other dichotomized measurements.

#### Context

This review considered all studies in sub-Saharan Africa conducted in institutional or community contexts.

#### Study design

This review considered quantitative studies, including randomized controlled trials, non-randomized controlled trials, before and after studies and interrupted time-series studies. We also considered analytical observational studies including prospective and retrospective cohort studies, case-control studies and analytical cross-sectional studies for inclusion.

#### Publication type

All studies published in the English language were considered for inclusion regardless of the date of publication.

### Exclusion criteria

Pilot or feasibility studies (studies aimed at assessing the feasibility of trials) were excluded from the review.

### Search strategy

We conducted a three-step search to locate both published and unpublished studies. First, we conducted an initial limited search in PubMed and CINAHL (EBSCO) to identify articles on the topic. We used the text words and index terms found in the articles to develop a full search strategy. Finally, we screened the reference list of all studies included in the systematic review to identify additional studies. The databases used in the final search were PubMed, CINAHL, SCOPUS, and ProQuest Databases of Dissertation and Theses (PQDT). The last search date for all databases was 16 September 2024 (Supplementary file S2).

### Study selection

We collated the retrieved references using EndNote citation software (Gotschall 2023). After removing duplicates, two reviewers screened the references for eligibility against inclusion criteria. GTF, RI and MS independently screened the articles for inclusion. Potentially relevant articles were retrieved in full. All disagreements that arose between the reviewers at each stage of the selection process were resolved through discussion.

### Assessment of methodological quality

Eligible studies were critically appraised by two independent reviewers using standardized critical appraisal instruments from the Joanna Briggs Institute (JBI) for experimental, quasi-experimental and observational studies. G.T.F., R.I., and M.S. appraised the methodological qualities of the studies. While there was a plan to invite a third reviewer in case of disagreements, all disagreements that arose during the appraisal process were resolved through discussion. All appraised studies were included in the review regardless of their methodological quality.

### Data extraction and synthesis

We extracted data from studies included in the review using an *a priori* prepared data extraction tool. We extracted data on study ID, study design, country, study participants, intervention, comparators, and outcome. We characterized the population, interventions, comparators, and outcome measures using tables. We pooled findings from the studies using the JBI System for the Unified Management of the Assessment and Review of Information (SUMARI) meta-analytic software (Piper 2019). For continuous outcomes, we calculated standardized mean difference (SMD) and their 95% confidence intervals. For dichotomous outcomes, we estimated relative risks (RR) and their respective 95% confidence intervals. Since our primary interest was to draw conclusions beyond those studies included in this review, by default, we considered random effects model. For dichotomous outcomes, we used restricted maximum likelihood estimation to estimate effect sizes. For continuous outcomes, we used inverse variance method of estimation. However, random effects model is not generally recommended if the number of studies to be pooled is less than five (Tufanaru et al. 2015, Partlett and Riley 2017, Deeks et al. 2019). Therefore, we used fixed effects model whenever the studies included in the meta-analysis are less than five. We assessed the presence and severity of statistical heterogeneity across studies using the standard chi squared and *I*² statistic. We explored sources of heterogeneity using visual evaluation of the confidence intervals in the forest plots, and subgroup analysis by subgroups of population and intervention types. We conducted sensitivity analysis by intervention type and source of heterogeneity to improve robustness of the analysis. For studies reporting on multiple interventions, where appropriate, we pooled the effect sizes separately. We used funnel plots to assess publication bias. However, we did not present the results here since the number of studies included in each meta-analysis is <10 and the analysis is expected to be underpowered (Afonso et al. 2024).

### Assessing certainty in the findings

We created determined certainty of evidence and created a Summary of Findings (SoF) using the Grading of Recommendations, Assessment, Development and Evaluation approach and GRADEpro GDT (McMaster-University-and-Evidence-Prime 2025).

## Results

**Figure 1.**
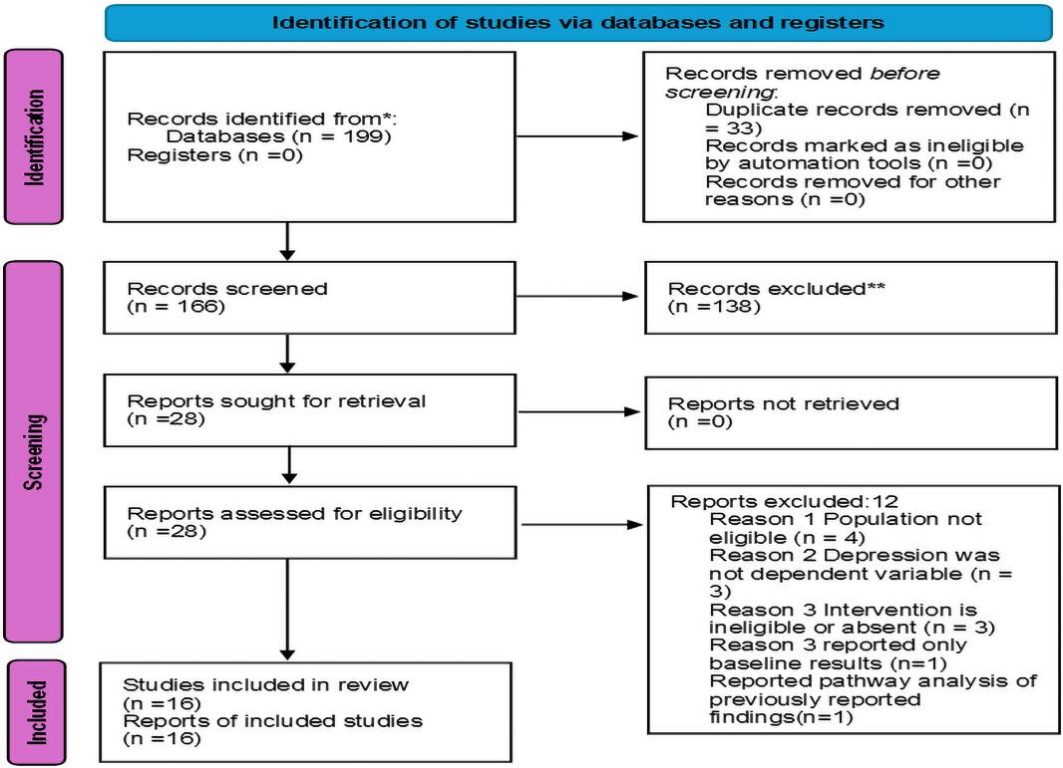
Study selection. Source: Page et al. (2021).

The initial search yielded 199 studies. After removing 33 duplicates, 166 studies were left for screening by title and abstract. Out of these, 28 were screened full text (Fig. 1). Twelve studies (Rotheram-Borus et al. 2011, 2015, Tsai et al. 2014, Tomlinson et al. 2015, 2018, Bryant et al. 2017, Christodoulou et al. 2019, Gureje et al. 2019, Nakku et al. 2021, Oyekunle et al. 2021, Spelke et al. 2022) were excluded by reasons (Supplementary file S3). The main reasons for exclusion after retrieval of full texts were ineligible study population (study population contained non-perinatal women), outcome of interest not reported as a dependent variable, ineligible intervention (intervention was lacking or was not provided by CHWs). In addition, one study was excluded because it reported only baseline results.

### Characteristics of the included studies

We included 16 articles in this review. Out of the studies included in this review, three of them (Cooper et al. 2009, Chibanda et al. 2014, Lund et al. 2020) used an individual randomized controlled trial design; nine of them (le Roux et al. 2013, Rotheram-Borus et al. 2014a, 2014b, 2023, Tomlinson et al. 2016, Baumgartner et al. 2021, Bliznashka et al. 2021, Comrie-Thomson et al. 2022, Kaaya et al. 2022) used a cluster randomized trial design; two studies (Le Roux et al. 2020, Kim et al. 2021) used a quasi-experimental study design and two studies (Stansert Katzen et al. 2020, Stansert Katzen et al. 2021) used a cohort study design. Only three of the included studies (Chibanda et al. 2014, Lund et al. 2020, Kaaya et al. 2022) focused on perinatal women with some depressive symptoms to explore the potential therapeutic effect of the interventions led by CHWs (Table 1). The remaining studies included perinatal women with and without depressive symptoms, mainly focusing on the preventive potential of the interventions led by CHWs (Table 2).

**Table 1.** Characteristics of included studies conducted among perinatal women with depression or depressive symptoms.

Study	Design	Country	Participant characteristics	Interventions/comparator	Results
Chibanda 2014 (Chibanda et al. 2014)	RCT	Zimbabwe	58 mothers with PND attending primary care at urban clinic (EPDS ≥ 11) and confirmed diagnosis of PND (DSM IV) at 6-week post-birth. Control: *n* = 28, mean age: 24 ± 4.4 years Intervention: *n* = 30, mean age 25 ± 5.4.	Control: Amitriptyline. Intervention: Weekly Group problem-solving therapy (PST) for 6 weeks	6 weeks post intervention (3-mo after birth) MD EPDS Score Amitriptyline: 8.22 ± 3.6 PST: 10.7 ± 2.7, *P* < 0.01.
Lund 2020 (Lund et al. 2020)	A double-blind individual RCT	South Africa	425 adult pregnant women with EPDS ≥ 13 from peri urban South Africa Control: *n* = 216, mean age = 27 (23–30) Intervention: *n* = 209, mean age = 27 (23–32)	Control: Enhanced usual care (EUC) (Routine ANC combined with 3 monthly phone calls) Intervention: A structured six-session psychological treatment conducted either at clinic or home	8-mo gestation Mean HDRS score EUC: 12.8 (4.53), *n* = 155 Intervention: 12.6(5.51), *n* = 133. MD (from baseline) in HDRS score EUC: −2.74 (−3.56 to −1.92) Intervention: −2.92 (−3.86 to −1.98) (RR = 0.97(0.86,1.09) 3-mo post-birth Mean HDRS score EUC:10.1(4.97), *n* = 187 Intervention: 9.1 (4.58), *n* = 148 MD HDRS score (change from baseline) EUC: −5.27 (−6.10 to −4.45) Intervention: −6.46 (−7.37 to −5.55) (RR = 0.89(0.79,1.0), *P* = 0.053. Mean EPDS Score EUC: 9.5 (5.70), *n* = 187 Intervention: 7.6 (5.20), *n* = 148. (RR = 0.78 (0.67 to (0.91), *P* = 0.001). Depressed (MINI), n (%) EUC: 34 (18.2), *n* = 187 Intervention: 23 (15.5), *n* = 148 (RR = 0.78 (0.45 to 1.33), *P* = 0.358) 12-mo post-birth Mean HDRS score EUC: 10.8 (5.07), *n* = 173 Intervention: 9.5 (4.32), *n* = 145 MD HDRS score (from baseline) EUC: −4.71(−5.65 to −3.76) Intervention: −6.01 (−6.95 to −5.08) (RR = 0.87(0.78,0.99), *P* = 0.028 HDRS recovery (HDRS < 8 at both 3-mo and 12-mo post-birth) EUC: 25 (14.5) Intervention: 31 (21.5) [RR = 1.49(0.92,2.4)], *P* = 0.10.
Kaaya 2022 (Kaaya et al. 2022)	CRCT	Tanzania	706 Pregnant women living with HIV with depression (PHQ-9 ≥ 9) in urban ANC clinics Control: *n* = 347, mean age = 29.5(5.3) Intervention: *n* = 359, mean age = 29.8(5.5)	Control: Enhanced usual care (identification and management of depression based on the WHO mental health gap depression treatment guideline for primary care settings). Intervention: Stepped care approach (Enhanced usual care plus the Healthy Options (prenatal group sessions of problem-solving therapy plus cognitive behavioral therapy	6-wk post-birth Mean PHQ-9 score Control: 6.9 (3.9) Intervention: 3.4 (4.2), adj MD = −3.56 (−4.55, −2.56), *P* < 0.01. Clinical diagnosis of MDD (PHQ-9 ≥ 9), *n* (%) Control: 111 (39.2%), *n* = 283 Intervention: 46 (13.5%), *n* = 341 (ARR 0.32(0.22, 0.47) *P* < 0.01. 9-mo post-birth Mean PHQ-9 score Control: 3.6 (3.4), Intervention: 2.6 (3.7), Adj MD = −1.03 (−1.86, −0.19), *P* < 0.05 Clinical diagnosis of MDD (PHQ-9 ≥ 9), *n* (%) Control: 34 (12.0), *n* = 283 Intervention: 31 (8.9), *n* = 348 ARR = 0.71 (0.40, 1.25) *P* = 0.28

ARR, adjusted risk ratio; EUC, enhanced usual care; DSM, Diagnostic and Statistical Manual; EPDS, Edinburg Postnatal Depression Scale; HDRS, Hamilton Depression Rating Scale; MD, mean difference; MDD, major depressive disorder; PHQ-9, Patient Health Questionnaire-9; PND, postnatal depression; PST, Problem Solving therapy; RCT, randomized controlled trial; RR, relative risk; HDRS recovery, the proportion of individuals with a HDRS <8 at both 3-months and 12-months post-birth.

**Table 2.** Characteristics of included studies conducted among perinatal women both with and without depressive symptoms.

Study	Design	Country	Participants	Intervention/comparator	Results
Kim 2021 (Kim et al. 2021)	Quasi-experimental	Kenya	417 Pregnant women and mothers of children under the age of 2. Control: *n* = 224, mean age = 26.59 (5.51). Intervention: *n* = 193, mean age = 26.14 (5.57)	Control: 14 sessions of home-based educational only intervention that focused on early childhood development over 7 months Intervention: Integrated Mothers and Babies Course and Early Childhood Development interventions (iMBC/ECD), 14− sessions of cognitive-behavioral, group-based intervention over 7 months.	8-mo post intervention PHQ-9 mean score Control: 2.42 (3.49), *n* = 183 Intervention: 3.21 (4.07), *n* = 157 MD (from baseline) PHQ-9 Control: −0.71 (4.29), *n* = 183 Intervention: −0.98 (5.48), *n* = 157. MD from baseline comparing intervention and control [MD = 0.1(−1.2, 1.4)] PHQ-9 dichotomized, none to mild (PHQ-9<9) Control: 176 (96.2%) Intervention: 139 (90.3%), *P* = 0.043. 14-mo after intervention Control: 2.86 (3.84), *n* = 183 Intervention: 2.85 (3.55), *n* = 157 PHQ-9 MD from baseline Control: −0.15 (4.63) Intervention: −1.43 (5.42) MD from baseline comparing intervention and control MD = −0.9 (−2.2, 0.4)] PHQ-9 dichotomized, none to mild (PHQ-9<9) Control: 169 (92.3%). Intervention: 146 (93.0%), *P* = 0.785
Baumgartner 2021 (Baumgartner et al. 2021)	Cluster randomized	Ghana	374 Pregnant women and mothers of children under the age of 2. Control: *n* = 153, mean age = 26.95 (6.53) Intervention: *n* = 221, mean age = 26.95 (6.99)	Control: 14 sessions of home-based educational intervention that only focused on early childhood development over 7 months Intervention: 14 sessions of home-based lay counselor-delivered, group-based Integrated Mothers and Babies Course and Early Childhood Development (iMBC/ECD) program	8-mo after intervention Mean PHQ-9 Control: 1.5 (0.8), *n* = 107 Mean change from baseline: −4.9 (−5.6, −4.2) Intervention: 1.9 (0.8), *n* = 159 Mean change from baseline: −4.1 (−4.9, −3.2) Mean change from baseline comparing intervention and control = (MD = −0.8 (−1.9, 0.3).
Comrie-Thomson 2022 (Comrie-Thomson et al. 2022)	Cluster randomized	Zimbabwe	457 Women who had given birth within 0–6 months and their male coparents. Control: *n* = 227, mean age = 25.1 (6.6) Intervention: *n* = 230, mean age = 25.8 (6.7)	Control: No treatment Intervention: Parental, health and gender empowerment intervention that encourages men’s support for the baby and for the women	12–15 mo-post intervention Mean EPDS Control: 4.0 (0.3) Intervention: 3.0 (0.4) ARR =0.7(0.5, 0.9), *P* < 0.01). Depression (EPDS > 12),n(%) Control: 18/219 (8.3%) Intervention: 19/199 (9.8%) AOR = 1.0 (0.6− 1.4), *P* = 0.81.
Cooper 2009 (Cooper et al. 2009)	RCT	South Africa	449 Pregnant women Control: *n* = 229 Intervention: 220	Control: Usual care (fortnightly visits by CHWs to assess physical and medical progress of mothers and infants Intervention: Home-based parenting guidance by health visitors to improve mother-infant relationships and security of infant attachment (two antenatal visits and weekly visits during the first 8 weeks of postpartum period.	6-month post-birth EPDS Score Control: 3.91 (5.80) Intervention: 2.78 (4.54) (*P* < 0.05). Depressive disorder (DSM-IV), n (%) Control: 29 (15.8) (*n* = 184) Intervention: 21 (12.4) (*n* = 170), n.s 12-mo post-birth EPDS score Control: 1.93 (4.54), *n* = 181 Intervention: 2.69 (5.86), *n* = 165 Depressive disorder (DSM-IV), n (%) Control: 28 (15.5) (*n* = 181) Intervention: 18 (10.9) (*n* = 165) No intervention effect on any of the outcomes at 12-mo post-birth.
Katzen 2021 (Stansert Katzen et al. 2021)	A cohort study	South Africa	1310 pregnant women attending ANC at clinics at deeply rural settings in the Eastern Cape of South Africa served by Philani home visits Control: *n* = 674, mean age (range) = 23 [14, 50] Intervention: *n* = 636, mean age (range) = 24 [14, 46]	Control: Usual care (free ANC and free HIV testing at clinics and hospitals, PMTCT, and support groups for PLHIV) Intervention: A comprehensive health and nutrition intervention through home visits (Philani): 6 antenatal and 5 postnatal visits between birth and 6 months post-birth)	12-mo post-birth Mean EPDS Control: 4.5(4.8) Intervention: 3.8(4.2) MD intervention vs control [MD = −0.8(−1.5, −0.1)]. Probable clinical depression (EPDS ≥18), n (%) Control : 30(7), *n* = 585 Intervention: 23(5.3), *n* = 565 [OR = 1(0.3,3.5) Depressed mood (EPDS >13), n (%) Control: 30(7), *n* = 585 Intervention: 23(5.3), *n* = 565. [OR = 0.8(0.4,1.6)]. Probable clinical Depression (EPDS >18), n (%) Control: 8(1.9), *n* = 565 Intervention: 8(18), *n* = 585. [OR = 1(0.3,3.5)].
Katzen 2020 (Stansert Katzen et al. 2020)			470 mothers giving birth at Zithulele Hospital and its 10 closest clinics, as well as mothers who had a home birth in the areas covered by the 10 clinics (Deeply rural setting). Control: *n* = 313, mean age = 24.9(7.6) Intervention: *n* = 157, mean age = 24.8(6.3)		3-mo post-birth Depression (EPDS > 13), *n* (%) Control: 15% (37/241) Intervention: 15% (19/124) 6-mo post-birth Depression (EPDS > 13), n(%) Control: 15% (37/250) Intervention: 12% (13/112) 9-mo post-birth Control: 12% (27/223) Intervention: 11% (13/117) 12-mo post-birth Control: 16% (33/209) Intervention: 9% (9/103) 24-mo post-birth Control: 8% (14/177) Intervention: 6% (5/85) A suggested trend for mothers in the HV group to show fewer depressive symptoms over time compared to SC (Estimate—0.55, SE—0.33, *P*-0.98).
Rotheram-Borus 2023 (Rotheram-Borus et al. 2023)	Cluster randomized	South Africa.	1238 Pregnant Women. Control: *n* = 594, mean age = 26.3 (5.6) Intervention: 644, mean age = 26.5 (5.5)		Difference of average log (EPDS +1) scores intervention-control Post-birth: −0.04(−0.17,0.10) 6-mo post-birth: 0.01(−0.13,0.15) 18-mo: −0.02 (−0.15,0.12) 3-year: −0.09 (−0.23,0.05) 5-year: −0.04(− 0.19,0.10) 8 years: −0.03 (−0.18,0.12) Odds ratio EPDS>13 Post-birth: 1.02(0.65,1.61) 6-mo: 1.24 (0.79,1.97) 18-mo: 1.06 (0.66,1.77) 3-year: 0.60 (0.38,1.00) 5-year: 1.00 (0.58,1.71) 8 years: 0.74 (0.37,1.55) Depression (EPDS >13) prevalence declined from 35.1% prenatally to 5.5% at 8-years, independent of the intervention.
LE Roux 2020 (Le Roux et al. 2020)	Quasi-Experimental	South Africa	1310 Pregnant women from rural eastern Cape attending ANC at clinics Control: *n* = 674, median age = 23 [14, 50] Intervention: *n* = 636, median age = 24 [14, 46]		6-mo post-birth Mean EPDS Score Control: 5.3(5.4) Intervention: 4.5(5) MD = −0.9 (−1.7, −0.2), *P* < 0.05 Depression (EPDS >13, n (%) Control: 47(9.9), *n* = 476 Intervention:30(6.3), *n* = 476 OR = 0.5 [0.3–1.1] Depression (EPDS >18, n (%) Control:18(3.8) *n* = 476 Intervention:14(2.9) *n* = 476. OR = 0.6 [0.2–1.8)
LE Roux 2013 (le Roux et al. 2013)	Cluster randomized		1238 Pregnant women Control: *n* = 594, mean age = 26.3 (5.6) Intervention: *n* = 644, mean age = 26.5 (5.5).	Control: Standard care in which PMTCT is provided in all clinics Intervention : Clinic-based support by HIV-positive peer Mentors plus standard care.	6-mo Among all women Not depressed (EPDS ≤ 13), *n* (%) Control: 413 (81.1), *n* = 509 Intervention: 444 (77.5), *n* = 573 OR = 0.80 (0.59, 1.08) Among PLHIV Not depressed ( EPDS ≤ 13), n (%) Control: 114 (73.1), *n* = 156 Intervention: 124 (72.1), *n* = 172 OR = 0.86 (0.57, 1.30)
Rotheram-Borus 2014b (Rotheram-Borus et al. 2014b)	Cluster randomized		1238 Pregnant women from peri urban setting South Africa Control: *n* = 594, mean age = 26.3(5.6) Intervention: *n* = 644, mean age = 26.5(5.5).		Post-birth, 6-mo and 18-mo Not depressed (EPDS ≤ 13), *n* (%) Control: 350(63.8), *n* = 549 Intervention: 357 (58.7), *n* = 608. OR = 0.81 (0.63, 1.05)
Tomlinson 2016 (Tomlinson et al. 2016)	Cluster randomized		1238 Pregnant women from peri urban setting South Africa Control: *n* = 594, mean age = 26.3(5.6) Intervention: *n* = 644, mean age = 26.5(5.5)		36 months post-birth Mean EPDS Control: 6.9(7.9) Intervention: 6.1(7.34) *P* < 0.01 Mean Hopkins Score Control: 36.2(17.4) Intervention: 34.2(14.9), *P* < 0.01
Rotheram-Borus 2014a (Rotheram-Borus et al. 2014a)	Cluster randomized	South Africa	1190 Pregnant WLH from peri urban setting South Africa Standard care: *n* = 656, mean age = 26.5(5.5) Intervention: *n* = 544, mean age = 26.5(5.5)		Not depressed (GHQ<7) 1.5-mo post-birth Standard care: 409 (87.8), *n* = 466 Intervention: 357 (94.7), *n* = 377 6-mo post-birth Standard care: 336(88.9), *n* = 378 Intervention: 285 (93.8), *n* = 304 12-mo post-birth Standard care: 152(84), *n* = 181 Intervention: 99 (93.4), 106 (OR = 1.08(1.03,1.13), *P* < 0.01).
Bliznashka 2021 (Bliznashka et al. 2021)	Cluster randomized	Tanzania	593 Pregnant women and mothers with a child. Control: *n* = 193, mean age = 26.5(6.5) CHW: *n* = 200, mean age = 27(5.7) CHW + CCT: *n* = 200, mean age = 27.1(6.7)	Control: No intervention Intervention group 1 (CHW): home visit responsive stimulation combined with health and nutrition intervention (Developmentally appropriate stimulation and promotion of caregiver responsiveness and sensitivity, combined with Health and nutrition interventions). Intervention group >2: CHW plus CCT (CHW + CCT).	9-mo after the intervention Mean HSCL-25 score(depressive subscale Control: 1.46 ± 0.46, *n* = 193 CHW: 1.05 ± 0.09, *n* = 200 CHW + CCT: 1.15 ± 0.27, *n* = 200 adj MD (CHW vs C) = −0.49 (−0.59, −0.38); *P* < 0.01 adj MD (CHW + CCT vs C) = −0.32 (−0.42, −0.22)], *P* < 0.01 18-mo after intervention Mean HSCL-25 score Control: 1.4 ± 0.43 CHW: 1.04 ± 0.13 CHW +CCT: 1.12 ± 0.19 adj MD (CHW vs C) = −0.43 (−0.56, −0.3), *P* < 0.01 and adj MD (CHW + CCT vs C) = −0.19 (−0.32, −0.07)]., *P* < 0.05

ARR, adjusted risk ratio; AOR, Adjusted Odds Ratio; CCT, conditional cash transfer; CHW, Community Health workers; DSM, Diagnostic and Statistical Manual; ECD, Early Childhood Development interventions; EPDS, Edinburg Postnatal Depression Scale; GHQ, General Health Questionnaire; HDRS, Hamilton Depression Rating Scale; HSCL-25, Hopkins Symptom Checlist-25; MD, mean difference; OR, odds ratio; PHQ-9: Patient Health Questionnaire-9; RCT, randomized controlled trial; RR, relative risk; HDRS recovery, the proportion of individuals with a HDRS <8 at both 3-month and 12-month postpartum.

Nearly 62.5% (10/16) of the studies (Cooper et al. 2009, le Roux et al. 2013, 2020, Rotheram-Borus et al. 2014b, 2023, Tomlinson et al. 2016 , Lund et al. 2020, Stansert Katzen et al. 2020, 2021) were conducted in South Africa. The remaining studies were conducted in Zimbabwe (Chibanda et al. 2014, Comrie-Thomson et al. 2022), Tanzania (Bliznashka et al. 2021, Kaaya et al. 2022), Ghana (Baumgartner et al. 2021), and Kenya (Kim et al. 2021). Seven studies(le Roux et al. 2013, Rotheram-Borus et al. 2014a, Rotheram-Borus et al. 2014b, Le Roux et al. 2020, Stansert Katzen et al. 2020, Tomlinson et al. 2020, Stansert Katzen et al. 2021, Rotheram-Borus et al. 2023) reported on one intervention using either different populations or different outcome measures, or different data points (Tables 1 and 2).

The studies included in this review used different data points to measure the outcomes. In addition, they employed different measurement scales, including Patient Health Questionnaire (PHQ-9), Edinburg Postnatal Depression Scale (EPDS), Hopkins Symptom Checklist-25 (HSCL-25), Hamilton Depression Rating Scale (HDRS), and General Health Questionnaire (GHQ). Among the studies that reported on dichotomized outcomes, one study used clinical DSM-IV criteria for the diagnosis of depression (Cooper et al. 2009) and one study (Lund et al. 2020) used Mini-International Neuropsychiatric Interview (MINI), the rest of the included studies used different cut off points of either of PHQ-9, EPDS, HDRS, or HSCL-25 scales. The nature, the dose, and the duration of follow up also varied across studies and so did the magnitudes of the effect estimates.

### Methodological qualities of the included studies

The appraisal scores for randomized trials ranged from 8/13 to 11/13. The main limitations of the randomized trials were the absence of blinding of participants, and/or intervention administrators, which is not feasible or challenging to implement in community-based behavioral interventions. Both quasi-experimental studies were rated 9/9. Both cohort studies were rated 9/11. No study was excluded based on appraisal score (Tables 3–5).

**Table 3.** Appraisal scores for individual and cluster randomized trials.

Study	Q1	Q2	Q3	Q4	Q5	Q6	Q7	Q8	Q9	Q10	Q11	Q12	Q13	Total
Kaaya 2022 (Kaaya et al. 2022)	Y	Y	Y	*N*	N	Y	N	Y	Y	Y	Y	Y	Y	10/13
Baumgartner 2021 (Baumgartner et al. 2021)	Y	Y	N	N	N	Y	N	Y	Y	Y	Y	Y	Y	10/13
Lund et al. (2020)	Y	Y	Y	N	N	Y	Y	Y	Y	Y	Y	Y	Y	11/13
Comrie-Thomson et al. (2022)	Y	Y	Y	N	N	Y	Y	Y	Y	Y	Y	Y	Y	11/13
Cooper et al. (2009)	Y	Y	Y	N	N	Y	Y	Y	Y	Y	N	Y	Y	10/13
Le Roux et al. (2013)	Y	Y	Y	N	N	Y	U	Y	Y	Y	N	Y	Y	9/13
Rotheram-Borus et al. (2023)	Y	Y	Y	N	N	Y	U	Y	Y	Y	N	Y	Y	9/13
Rotheram-Borus et al. (2014b)	Y	Y	Y	N	N	Y	U	Y	Y	Y	N	Y	Y	9/13
Tomlinson et al. (2016)	Y	Y	Y	N	N	Y	U	Y	Y	Y	N	Y	Y	9/13
Rotheram-Borus et al. (2014a)	Y	Y	N	N	N	Y	U	Y	Y	Y	N	Y	Y	8/13
Chibanda et al. (2014)	Y	Y	Y	N	N	Y	N	Y	Y	Y	N	Y	Y	9/13
Bliznashka et al. (2021)	Y	Y	Y	N	N	Y	Y	Y	Y	Y	Y	Y	Y	11/13

Y, yes; N, no; U, unclear.

Q1. Was true randomization used for assignment of participants to treatment groups?

Q2. Was allocation to treatment groups concealed?

Q3. Were treatment groups similar at the baseline?

Q4. Were participants blind to treatment assignment?

Q5. Were those delivering treatment blind to treatment assignment?

Q6. Were treatment groups treated identically other than the intervention of interest?

Q7. Were outcomes assessors blind to treatment assignment?

Q8. Were outcomes measured in the same way for treatment groups?

Q9. Were outcomes measured in a reliable way?

Q10. Was follow up complete and if not, were differences between groups in terms of their follow up adequately described and analyzed?

Q11. Were participants analyzed in the groups to which they were randomized?

Q12. Was appropriate statistical analysis used?

Q13. Was the trial design appropriate, and any deviations from the standard RCT design (individual randomization, parallel groups) accounted for in the conduct and analysis of the trial?

**Table 4.** Appraisal scores of quasi-experimental studies.

Study	Q1	Q2	Q3	Q4	Q5	Q6	Q7	Q8	Q9	Total
Kim 2021 (Kim et al. 2021)	Y	Y	Y	Y	Y	Y	Y	Y	Y	9/9
Le Roux 2020 (Le Roux et al. 2020)	Y	Y	Y	Y	Y	Y	Y	Y	Y	9/9

Y, yes; N, no; U, unclear.

Q1. Is it clear in the study what is the “cause” and what is the “effect” (i.e. there is no confusion about which variable comes first)?

Q2. Was there a control group?

Q3. Were participants included in any comparisons, similar?

Q4. Were the participants included in any comparisons receiving similar treatment/care, other than the exposure or intervention of interest?

Q5. Were there multiple measurements of the outcome, both pre and post the intervention/exposure?

Q6. Were the outcomes of participants included in any comparisons measured in the same way?

Q7. Were outcomes measured in a reliable way?

Q8. Was follow-up complete and if not, were differences between groups in terms of their follow-up adequately described and analyzed?

Q9. Was appropriate statistical analysis used?

**Table 5.** Appraisal scores of cohort studies.

Study	Q1	Q2	Q3	Q4	Q5	Q6	Q7	Q8	Q9	Q10	Q11	Total
Katzen 2021 (Stansert Katzen et al. 2021)	N	Y	Y	Y	Y	N	Y	Y	Y	Y	Y	9/11
Katzen 2020 (Stansert Katzen et al. 2020)	N	Y	Y	Y	Y	N	Y	Y	Y	Y	Y	9/11

Q1. Were the two groups similar and recruited from the same population?

Q2. Were the exposures measured similarly to assign people to both exposed and unexposed groups?

Q3. Was the exposure measured in a valid and reliable way?

Q4. Were confounding factors identified?

Q5. Were strategies to deal with confounding factors stated?

Q6. Were the groups/participants free of the outcome at the start of the study (or at the moment of exposure)?

Q7. Were the outcomes measured in a valid and reliable way?

Q8. Was the follow up time reported and sufficient to be long enough for outcomes to occur?

Q9. Was follow up complete, and if not, were the reasons to loss to follow up described and explored?

Q10. Were strategies to address incomplete follow up utilized?

Q11. Was appropriate statistical analysis used?

### Characteristics of the interventions

To describe the effect of the interventions within the context of their baseline levels of depression, we categorize the interventions into preventive and therapeutic categories. For the sake of this review, we considered interventions that targeted only perinatal women with depression or depressive symptoms as therapeutic interventions. We considered interventions that targeted all perinatal women regardless of their level of depression scores as prevention interventions.

- Therapeutic interventions

Three of the studies included in this review (Chibanda et al. 2014, Lund et al. 2020, Kaaya et al. 2022) focused on perinatal women with some depressive symptoms to explore the potential therapeutic effect of interventions led by CHWs. Generally, therapeutic interventions reported by the studies included in this review include counseling and problem-solving therapy conducted either at individual level or group level.

Group-based interventions were effective at least in the short term. Interestingly, Chibanda 2014 (Chibanda et al. 2014) reported that structured group-based problem solving therapy delivered by CHWs was significantly effective in reducing perinatal depression compared to pharmacotherapy (amitriptyline) at 6 weeks postpartum. Six weeks after the intervention, the drop in mean EPDS score was greater in the problem-solving therapy group (MD = 8.22 ± 3.6) compared to the amitriptyline group (10.7 ± 2.7); *P* < 0.01.

In addition, as demonstrated by Kaaya et al. (2022), group-based problem-solving therapy has short-term effects of reducing perinatal depression and depressive symptoms. Kaaya et al. (2022) evaluated the effect of stepped care model for the management of depression using evidence-based group problem solving and cognitive behavioral therapy among perinatal women living with HIV with depression (PHQ-9 ≥ 9. In the treatment arm, the problem-solving therapy was delivered during prenatal care, and the cognitive behavioral therapy was provided for those women showing depressive symptoms postnatally at 6 weeks. The enhanced usual care (control arm) comprised training on the identification and management of depression based on the WHO mental health gap depression treatment guideline for primary care settings. Compared to the enhanced usual care, the stepped care model for the management resulted in statistically significant reduction in the prevalence of major depressive disorder (MDD) (PHQ-9) and depressive symptoms (PHQ-9 scores) 6 weeks after birth even though the effect was not statistically significant at 9 months. At 6 weeks postpartum, women in the stepped care intervention had a 68% [RR 0.32(0.22, 0.47)] lower likelihood of MDD (PHQ-9 ≥ 9) (*P* < 0.05) and significantly lower mean PHQ-9 scores (MD = −3.56 (−4.55, −2.56) than women in enhanced usual care group (*P* < 0.01). The effect was not statistically significant 9 months after birth (Table 1).

Study conducted in South Africa by Lund 2020 evaluated the effectiveness of clinic-based structured six counseling sessions of weekly psycho-social treatment of perinatal depression among women with EPDS score ≥13 (Lund et al. 2020). The study reported effect estimates varying not only by data points but also by the type of measurement scale (EPDS vs HDRS). The study reported inconsistent findings. The authors found no significant differences in response on the HDRS (Hamilton Depression Rating scale) between the intervention and control arm (routine antenatal care) at 8 months gestation [RR = 0.97(0.86,1.09)] and 3 months postpartum [RR = 0.89(0.79,1.0)]. There was statistically non-significant difference [RR = 0.78 (0.45 to 1.33), *P* = 0.358] in the risk of depression (depression diagnosis using the MINI scale) between the two arms at 3 months postpartum. However, there was a 22% [RR = 0.78 (0.67 to 0.91), *P* = 0.001] higher reductions in EPDS score among intervention participants when compared to control participants at 3 months postpartum. At 12 months post-birth, the reduction in HDRS score was 13% higher (RR = 0.87(0.78,0.99) among the intervention arm when compared to the control arm. Nevertheless, the HDRS recovery (proportion of individuals with a HDRS <8 at both 3-month and 12-month postpartum) of the intervention participants was not significantly different from the control arm [RR = 1.49(0.92,2.4)] (Table 1).

(II) Prevention interventions

Overall, the preventive interventions have addressed psycho-social issues of perinatal women through behavioral, health and nutrition components. One of the prevention interventions reported in the studies included in this review was conducted among women living with HIV. Compared to the standard care (free Prevention of Mother to Child Transmission (PMTCT) at clinics, home-based peer support intervention resulted in statistically significant increment in the proportion of women who are not depressed (GHQ < 6), with effects lasting up to 12 months after birth (OR = 1.08, *P* < 0.01) Rotheram-Borus 2014a (Rotheram-Borus et al. 2014a)

The remaining studies targeted all perinatal women. These include:

- Home visit containing responsive stimulation combined with health and nutrition intervention with, and without cash transfer (Bliznashka et al. 2021);
- Parenting intervention that encourages men’s support for the baby and for the mother (Comrie-Thomson et al. 2022);
- Home-based parenting guidance aimed at sensitizing the mother to respond to the need of the baby and secure mother-infant attachment (Cooper et al. 2009);
- A home-based bimonthly intervention integrating maternal and child mental health intervention (Baumgartner et al. 2021, Kim et al. 2021).
- A comprehensive maternal and child health and nutrition intervention through home visits (I. M. le Roux et al. 2013, Rotheram-Borus et al. 2014b, Tomlinson et al. 2016, Le Roux et al. 2020, Stansert Katzen et al. 2020, Stansert Katzen et al. 2021, Rotheram-Borus et al. 2023).

The first category of intervention, responsive stimulation combined with health and nutrition intervention was associated with statistically significant reduction in depressive symptoms (HSCL-25 scores) both at 9 months and 18 months after the intervention (Bliznashka et al. 2021). The intervention was significant both with and without conditional cash transfer ($4.30 for ANC visits and $2.2 for child monitoring visits) (Table 2).

The second category of intervention was a parenting intervention that was aimed at enhancing men’s support for the mother and for the baby (Comrie-Thomson et al. 2022) Compared to the control arm with no intervention, the gender synchronized intervention was effective in reducing depressive symptoms 12–15 months after the intervention even though the effect on the dichotomized outcome (the proportion of mothers with EPDS ≥ 12) was not significant. Participants exposed to the gender synchronized parenting intervention had a 30% [aRR = 0.7 (0.5, 0.9), *P* < 0.01] greater reduction in depression scores (EPDS score). However, the effect of the intervention on the dichotomized outcome [clinically significant depression (EPDS ≥ 12)] was not significant [aOR = 1.0 (0.6, 1.4), *P* = 0.81] 12–15 months after the intervention (Comrie-Thomson et al. 2022).

The third category of intervention is parenting guidance. In the study by Cooper et al. (2009), compared to usual care (fortnightly visits by CHWs to assess physical and medical progress of mothers and infants), there was a statistically significant effect of home-based parenting guidance (delivered during late pregnancy and the first 6 months after birth) in reducing depressive symptoms at 6 months postpartum follow up but not at 12 months postpartum. The intervention was aimed at sensitizing the mother to respond to the need of the baby and secure mother-infant attachment. The effect of the intervention on reducing the proportion of mothers with clinical depression (based on DSM-IV criteria) was not statistically significant at 6 months or 12 months postpartum.

Compared to an early childhood educational (EC-only) intervention, an integrated mother-child baby course (IMC) combined with early childhood development (IMC + EC) did not significantly reduce depressive symptoms both in Kenya and Ghana (Baumgartner et al. 2021, Kim et al. 2021) (Table 2).

Another intervention with a relatively longer period of follow-up is a comprehensive maternal and child health and nutrition intervention. Seven studies (le Roux et al. 2013, Rotheram-Borus et al. 2014b, Tomlinson et al. 2016, Le Roux et al. 2020, Stansert Katzen et al. 2020, Stansert Katzen et al. 2021, Rotheram-Borus et al. 2023) evaluated the effect of the Philani intervention (home visits addressing general maternal and child health, HIV, tuberculosis, alcohol use, and nutrition) even though they used different populations, or reported the effects of the intervention using different data points and outcomes measures. Compared to usual care in which maternal and child health care is provided free of charge at clinics, the home-visiting (Philani) intervention significantly reduced depressive symptoms (EPDS scores) at 6 months (Le Roux et al. 2020) but did not significantly increase the proportion of individuals who are not depressed EPDS < 13 (le Roux et al. 2013). The effect of Philani home visits on the prevalence of depressed mood (EPDS >13) was significant only 3 years after birth. Three years after birth, intervention participants had a 30% [OR = 0.6(0.38,1.0)] reduced risk depressed mood (EPDS >13) when compared to control participants (Rotheram-Borus et al. 2023).

There was no significant difference of mean log (EPDS +1) between Philani home visits and standard care at 2 weeks, 6-month, 18 month, 5 years, and 8 years after birth even though the difference was significant 3 years after birth (Tomlinson et al. 2016). Three years after birth, significant effect of the intervention was recorded both on the dichotomous outcome (EPDS >13) (Rotheram-Borus et al. 2023) and continuous outcomes (Hopkins and EPPDS scores) (Tomlinson et al. 2016). Hence, the effectiveness of comprehensive health and nutrition interventions through home visits (Philani intervention) is not conclusive (Table 2).

### Meta-analytic findings

- Prevention interventions

Dichotomous outcomes

**Figure 2.**
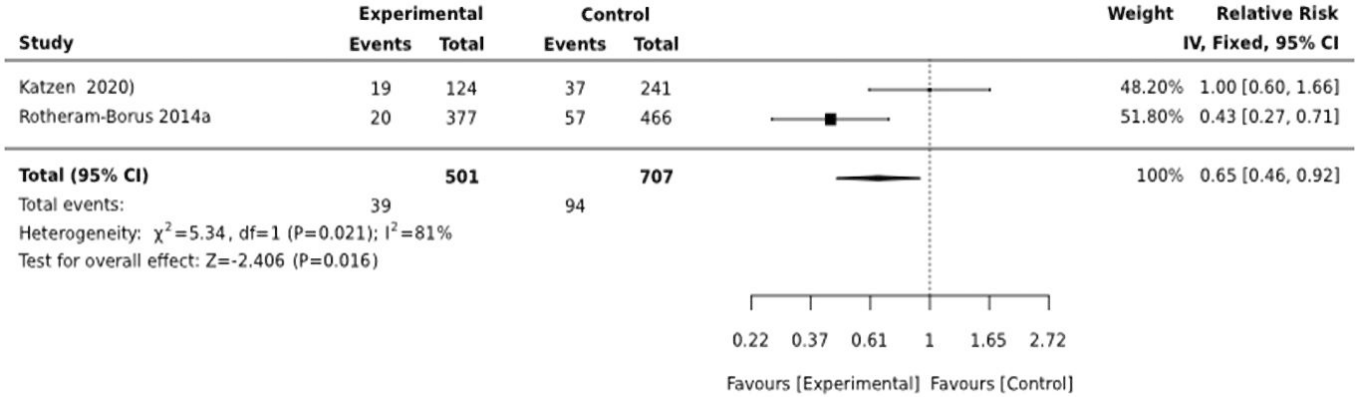
Effect of preventive psycho-social interventions on depressed mood during the first three months of birth.

During the first 3 months after birth, CHW-led interventions reduced the risk of depressed mood by 35% [RR = 0.65(0.46,092)] (Fig. 2).

**Figure 3.**
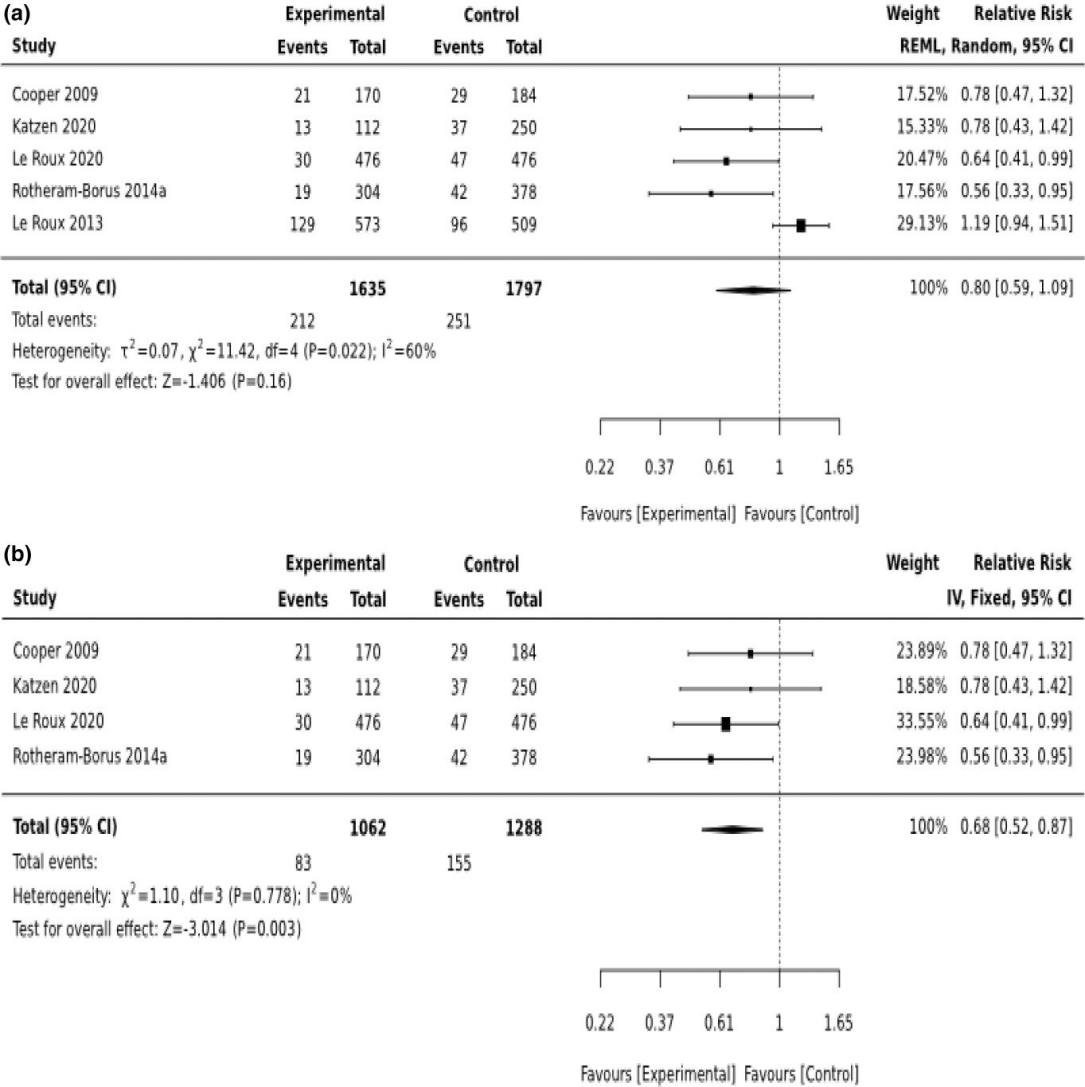
(A) Initial analysis to estimate preventive effect of psycho-social interventions on depressed mood 6-months post-birth. (B) Final robust analysis to estimate preventive effect of psycho-social interventions on depressed mood 6-months post-birth.

At 6 months post-birth, there was heterogeneity of effect (*I*^2^ = 72) (Fig. 3a). After conducting sensitivity analysis, we learned that one study (le Roux et al. 2013) contributed to the heterogeneity. Excluding the study from the model completely removed the heterogeneity (*I*^2^ = 0) (Fig. 3b). All the studies included in the estimation of the effect on the incidence of depressed mood at 6-months post-birth are from South Africa. Three of them focus on the effect of Philani intervention in different populations. Overall, the meta-analysis shows that the intervention significantly reduced the risk of depressed mood by 32% 6-months post-birth [RR = 0.68(0.52, 0.87)] (Fig. 3b).

**Figure 4.**
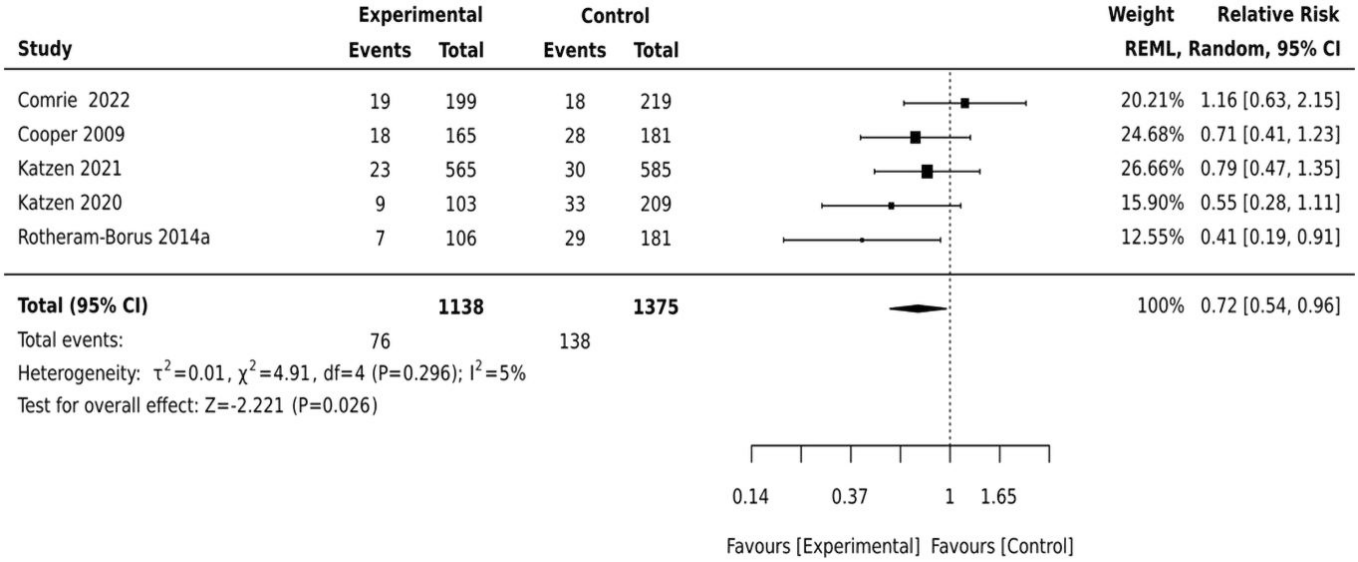
The effect of preventive psycho-social intervention on depressed mood 9–12 months after birth.

The preventive psycho-social interventions reduced the risk of depressed mood by 38% [RR = 0.72(0.54,0.96)] 9–12 months after birth (Fig. 4).

Continuous outcomes

**Figure 5.**
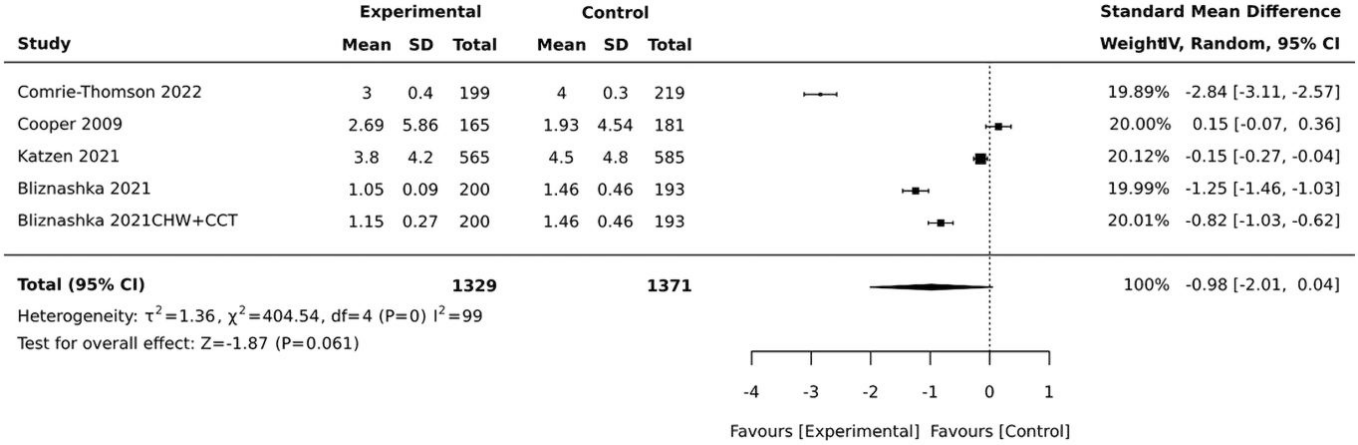
The effect preventive psycho-social interventions on continuous outcomes (depressive symptoms) 9–12 after birth.

Even though not statistically significant, CHW-led interventions reduced depressive symptoms 9–12 months post-birth [SMD = −0.98(−2.01,0.04)] (Fig. 5).

**Figure 6.**
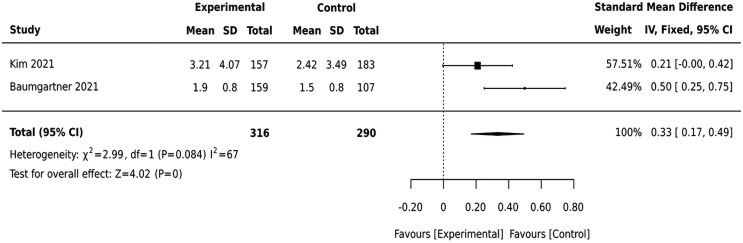
Integrated child course combined with early childhood development educational intervention (IMC + EC) vs EC only educational intervention.

We analyzed the findings from two studies separately because of their difference in the comparators used. The studies compared an early childhood educational (EC-only) intervention with an integrated mothers and baby course (IMC) combined with early childhood development (IMC + EC) (Baumgartner et al. 2021, Kim et al. 2021). Surprisingly, the pooled effect of the intervention from two studies tends to favor the intervention that only focused on early childhood development, as opposed to the integrated maternal and child mental health intervention addressing both maternal mental health and early childhood components [SMD = 0.33(0.17,049)] (Fig. 6).

(B) Therapeutic interventions

**Figure 7.**
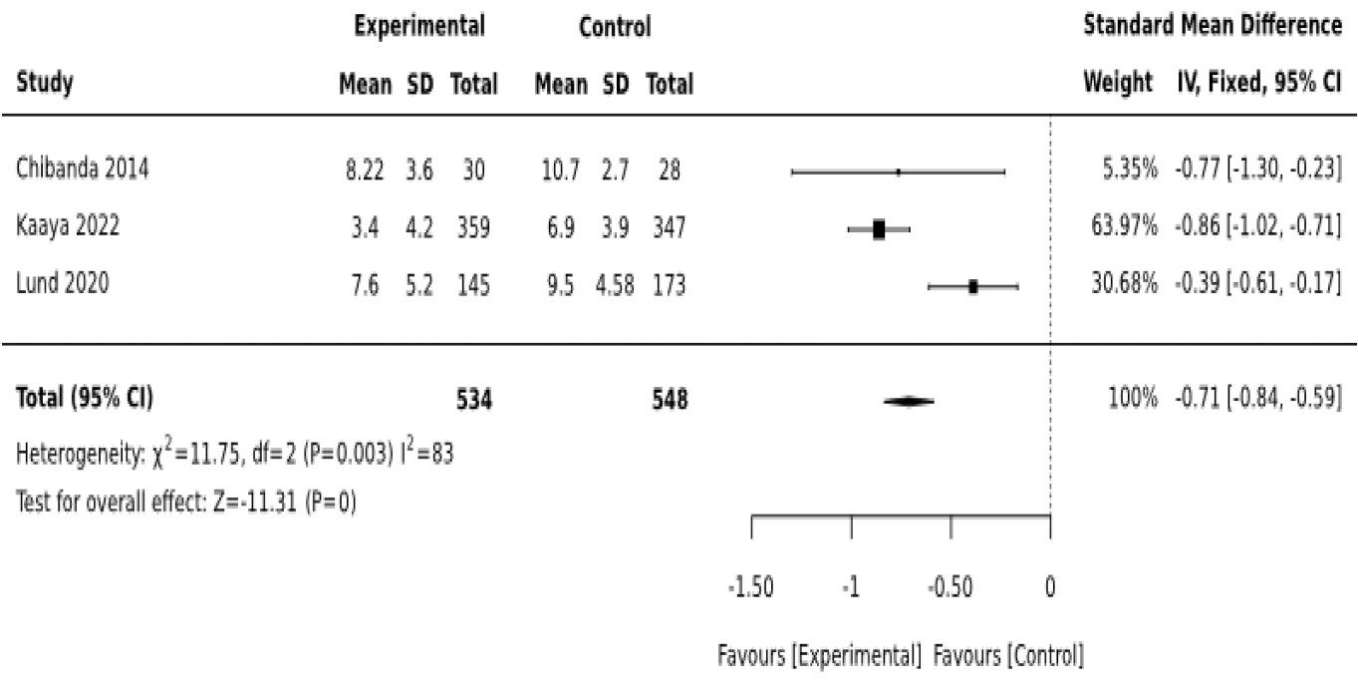
Therapeutic effect of CHWs-led psycho-social interventions in reducing depressive symptoms among mothers with moderate depressive symptoms (first 3-months after birth).

**Figure 8.**
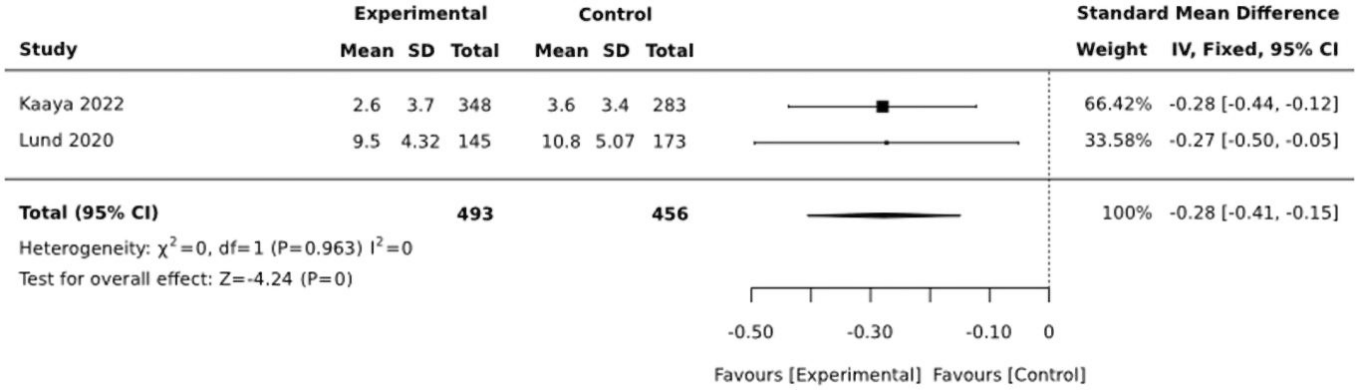
Therapeutic effect of CHWs-led psycho-social interventions in reducing depressive symptoms among mothers with moderate depressive symptoms (9–12 months post-birth).

Psycho-social interventions led by CHWs also appear to be effective in reducing depressive symptoms among women with perinatal depression. The interventions resulted in significant reduction in depressive symptoms during the first 3 months [SMD = −0.71 (−0.84, −0.59)] (Fig. 7). The effect of the intervention lasts 9–12 months after birth [SMD = −0.28 (−0.41, −0.15)] (Fig. 8).

### Summary of findings

#### Preventive interventions

In the first 3 months of birth, CHW-led interventions result in 47 fewer cases of women with depressed mood (per 1000 women) compared to usual care [Low quality evidence]. Six months after birth, CHWs-led preventive psycho-social interventions result in 24 fewer cases of perinatal women with depressed mood compared to the usual care per 1000 perinatal women (low quality evidence). The effect is sustained through 9–12 months post-birth resulting in 28 fewer cases of depressed mood per 1000 women (low quality evidence). Psycho-social interventions delivered by CHWs also lower depressive symptoms by 0.6 standard deviation (SD) units 9–12 months after birth (very low-quality evidence) (Table 6).

**Table 6.** Summary of findings for preventive psycho-social interventions compared to usual care.

Certainty assessment							№ of patients		Effect		Certainty	Importance
№ of studies	Study design	Risk of bias	Inconsistency	Indirectness	Imprecision	Other considerations	Psychosocial Preventive Intervention	Usual Care	Relative (95% CI)	Absolute (95% CI)		
												
Depressed mood (follow-up: mean 12 months)												
5	randomized trials	serious^a^	serious^b^	not serious	not serious	none	76/1136 (6.7%)	138/1378 (10.0%)	RR 0.72 (0.54 to 0.96)	28 fewer per 1000 (from 46 fewer to 4 fewer)	⨁⨁◯◯ Low^a,b^	
Depressive symptoms (follow-up: mean 12 months)												
4	randomized trials	serious^a^	serious^b^	not serious	serious^c^	none	1329	1371	—	SMD 0.6 SD lower (0.68 lower to 0.52 lower)	⨁◯◯◯ Very low^a,b,c^	
Depressed mood (6) (follow-up: mean 6 months)												
4	non-randomized studies	serious^a^	serious^b^	not serious	not serious^d^	none	83/1062 (7.8%)	155/1288 (12.0%)	RR 0.80 (0.59 to 1.09)	24 fewer per 1000 (from 49 fewer to 11 more)	⨁⨁◯◯ Low^a,b,d^	
Depressed Mood (follow-up: mean 3 months)												
2	randomized trials	serious^a,e^	serious^b^	not serious	not serious	none	39/501 (7.8%)	94/707 (13.3%)	RR 0.65 (0.46 to 0.92)	47 fewer per 1000 (from 72 fewer to 11 fewer)	⨁⨁◯◯ Low^a,b,e^	

CI, confidence interval; RR, risk ratio; SMD, standardized mean difference.

Explanations

^a^Some findings come from high quality observational studies.

^b^Inconsistencies in the effectiveness of the interventions across studies.

^c^Wide confidence interval

^d^The confidence interval of the pooled estimate is wide and overlaps the line of no effect.

^e^Lack of blinding of study participants, outcome assessors and those delivering the intervention.

An integrated maternal and child health course (IMC) combined with early childhood educational (EC) intervention is not better than an EC only intervention. The average depressive symptoms in the combined intervention (IMC + EC) arm are 0.33 SD higher compared to the EC only educational intervention arm (moderate-quality evidence) (Table 7).

**Table 7.** Summary of findings for integrated maternal and child behavioral intervention versus early childhood-focused education only intervention.

Certainty assessment							№ of patients		Effect		Certainty	Importance
№ of studies	Study design	Risk of bias	Inconsistency	Indirectness	Imprecision	Other considerations	Integrated MCH Behavioral Intervention	Early childhood focused Education only Intervention	Relative (95% CI)	Absolute (95% CI)		
Depressive Symptoms (follow-up: mean 8 months)												
2	non-randomized studies	serious^a^	not serious	not serious	not serious	none	316	290	—	SMD 0.33 SD higher (0.17 higher to 0.49 higher)	⨁⨁⨁◯ Moderate^a^	

CI, confidence interval; SMD, standardized mean difference.

Explanations

^a^Studies did not blind participants and intervention administrators.

#### Therapeutic interventions

Moderate-quality evidence indicates that psycho-social therapeutic interventions delivered by CHWs lower depressive symptoms by an average of 0.71 standard deviation (SD) units in the first 3 months of birth and 0.28 SD units 9–12 months after birth among perinatal women with moderate depressive symptoms (Table 8) (Box 1).

Box 1. How effective are CHWs-led interventions in reducing the risk of depressed mood and depressive symptoms during perinatal period? Effectiveness of CHWs in reducing the risk of depressed mood

- In the first 3 months of birth, CHW-led interventions result in 47 fewer women with depressed mood (per 1000 perinatal women) compared to usual care (low quality evidence).
- Six months after birth, the interventions result in 24 fewer women with depressed mood compared to the usual care per 1000 perinatal women (low quality evidence).
- Compared to usual care, CHW-led psycho-social interventions result in 28 fewer women with depressed mood per 1000 women 9–12 months post-birth (Low quality evidence).
- An integrated maternal and child health course (IMC) combined with early childhood educational (EC) intervention is not superior to an EC-only intervention (moderate quality evidence).

Effectiveness of CHWs in reducing depressive symptoms

- Psycho-social therapeutic interventions delivered by CHWs lower depressive symptoms by an average of 0.71 standard deviation (SD) units in the first three months of birth and 0.28 SD units 9–12 months after birth among perinatal women with moderate depressive symptoms (moderate quality evidence).

**Table 8.** Summary of findings for psycho-social therapeutic interventions compared to usual care.

Certainty assessment							№ of patients		Effect		Certainty	Importance
№ of studies	Study design	Risk of bias	Inconsistency	Indirectness	Imprecision	Other considerations	Psychosocial therapeutic interventions	Usual care	Relative (95% CI)	Absolute (95% CI)		
Depressive Symptoms (follow-up: mean 3 months)												
3	randomized trials	serious^a^	not serious	not serious	not serious	none	534	548	—	SMD **0.71 SD lower** (0.84 lower to 0.59 lower)	⨁⨁⨁◯ Moderate^a^	
Depressive Symptoms (follow-up: mean 12 months)												
2	randomized trials	serious^b^	not serious	not serious	not serious	none	493	456	—	SMD **0.28 SD lower** (0.41 lower to 0.15 lower)	⨁⨁⨁◯ Moderate^b^	

NB:Point estimates are written in bold. CI, confidence interval; SMD, standardized mean difference.

Explanations

^a^The studies did not blind participants, intervention administrators, and outcome assessors.

^b^The studies did not blind participants and intervention administrators.

## Discussion

This review attempted to provide answers to how effective CHWs are in managing and preventing perinatal depression in sub-Saharan Africa. The interventions are reported in this review broadly categorized into therapeutic and prevention interventions. Majority of the studies reported on prevention interventions, indicating major gap in studies reporting on therapeutic interventions.

- Therapeutic interventions

Moderate-quality evidence indicates that psycho-social therapeutic interventions delivered by CHWs lowers depressive symptoms by 0.71 standard deviation (SD) units in the first 3 months of birth and 0.28 SD units 12 months after birth among mothers diagnosed with moderate perinatal depression. This finding is encouraging to the continent as it struggles with the shortage of trained human power. Interestingly, Chibanda et al. (2014) reported that group problem solving therapy delivered by CHWs was significantly effective in reducing perinatal depression compared to pharmacotherapy (amitriptyline). This supports the argument that states that psychological treatment should be the first line of treatment for perinatal depression (Cuijpers et al. 2023). On the other hand, the main limitation of this finding is that it comes only from three studies. In addition, it is not clear whether the reductions in the depressive symptoms were clinically meaningful. None of the studies in this category used dichotomized measures to assess the clinical meaningfulness of the interventions.

Prevention interventions

Very low-quality evidence indicates that psycho-social interventions delivered by CHWs results in 25 fewer cases of depressed mood per 1, 000 women 6 months after birth. Low quality evidence indicates that the effect of CHWs-led psycho-social intervention is sustained through 9–12 months after birth resulting in 28 fewer cases of depressed mood per 1000 women.

Given the overall quality of evidence, there is a need for contextual considerations. This underscores the importance of conducting further analysis on the feasibility, applicability and meaningfulness of the interventions while adapting the interventions to the local contexts. As described earlier, most studies included in this review originate from South Africa. It also appears that most interventions conducted in South Africa are relatively not effective. For instance, the effect of Philani intervention, which had the longest duration of follow up seems to be inconclusive (Tomlinson et al. 2016, Rotheram-Borus et al. 2023). While there might be other reasons for this, the lack of statistically significant effect might have been because depression scores significantly reduced in both arms. Depression declined from 35.1% prenatally to 5.5% 8 years after birth. Other studies from South Africa also reported statistically non-significant effects (Cooper et al. 2009, Lund et al. 2020).

On the other hand, it is encouraging to see that some interventions, such as peer supports are effective both for prevention and management of perinatal depression, even in South Africa. For instance, in the study by Rotheram-Borus et al. (2014a), peer supports significantly reduced the incidence of depressed mood among women living with HIV with effects lasting through 12 months [OR = 1.08(1.03,1.13)] (Rotheram-Borus et al. 2014a). This agrees with emerging global evidence from high income countries which indicates that peer support interventions could improve perinatal depression (Shah et al. 2024).

Most studies conducted outside South Africa consistently reported statistically significant effect of CHW-led interventions both for prevention and management of perinatal depression. For instance, study conducted in Tanzania reported that responsive stimulation combined with health, and nutrition components is effective both with and without conditional cash incentives (Bliznashka et al. 2021). This study clearly indicated that the risk of depressed mood among perinatal women might be reduced by indirectly by addressing health and nutrition components and developmentally appropriate childhood stimulation. Relatedly, there might be potential for interventions focused on early childhood development. The effect of integrated maternal and child course (IMC) combined with early childhood educational intervention was not significantly different from an educational only intervention that focused on early childhood development (Baumgartner et al. 2021, Kim et al. 2021). This might imply that targeting early childhood development is sufficient intervention to reduce perinatal depression by itself. Interestingly, the pooled effect favors early childhood focused education only intervention. Even though the finding is surprising, it may also indicate that strengthening parenting skills is effective even in the absence of direct interventions for perinatal depression. Even though not included in this review (because the intervention targeted mother-child dyads beyond 12 months of postnatal period), a study from Uganda found significant effect of integrated child development and maternal psychological wellbeing program on depressive symptoms measured by the Center for CES-D (Singla et al. 2015). Hence, further studies are required to explore the potential benefits of interventions that focus early childhood in reducing the risk of perinatal depression.

While interpreting the findings reported in this review, it is essential to note that large sample sizes are required to detect differences in effect sizes. As acknowledged in the studies included in this review (Baumgartner et al. 2021, Kim et al. 2021), both low clinical sensitivity among the non-depressed population and the low power of small studies contribute to statistically non-significant effects of these interventions. A similar finding was reported from a systematic review of global evidence that highlighted that psycho-social interventions have little effect among non-depressed perinatal women (Martín-Gómez et al. 2022).

While research from sub-Saharan Africa on perinatal mental health is generally lagging, if attention is given to perinatal mental health, the region could avert undesirable consequences of perinatal depression such as higher risk of preterm birth, low birth weight, and other adverse birth outcomes (Dadi et al. 2022). With the presence of strong social cohesion, sub-Saharan Africa is known to offer strong resources to ensure the mental health and wellbeing of perinatal women. However, the presence of cultural prejudices remain obstacles toward health seeking behavior for mental health problems during the perinatal period (Monaghan et al. 2021). Hence, CHWs may play a substantial role in bridging this gap in mental healthcare utilization especially by demystifying misperceptions and prejudices related to mental health (Sakina et al. 2022). CHWs have played a critical role in increasing access to primary healthcare services and supplementing the formal health care system (Patel and Nowalk 2010, Woldie et al. 2018, Ludwick et al. 2020). There is a potential opportunity to use CHWs for the prevention and management of perinatal depression because the work of CHWs in many settings in sub-Saharan Africa is integrated as part of the primary healthcare system to provide a comprehensive and sustainable services (Mupara et al. 2023). Even though their contribution to perinatal mental health services could even be more critical, there is still scarcity of evidence on interventions that are feasible, applicable, meaningful and effective within the context of sub-Saharan Africa. Hence, the optimal delivery format of perinatal mental health interventions is not clear from the currently available evidence (Ng'oma et al. 2020).

While findings in the current review might be used with further contextual considerations, there is a need for additional high-quality trials to develop or adapt culturally acceptable and locally feasible interventions. Future trials and implementation projects should address some limitations of the studies reported in the current review. Among important considerations are implementation fidelity of the interventions. For instance, among the studies that reported non-significant effect estimates, Kim et al. (2021) reported consistently fewer depressive symptoms among women with higher rates of program attendance. Lower attendance was also an issue in the study by Lund et al. (2020), which reported only 53.3% attendance. This underscores the fact that future studies need to implement intensive methods and strategies to retain study participants, and intensive training, monitoring, and supervision to enhance adherence to the intervention protocol. Hence, there is a need for careful logistical and skill arrangements for the adaptation. This involves preparation of training curriculum and cascading the training. The experiences of cascade approach of training that has been shown to be effective in integrating mental health at primary healthcare setting might be modified and adapted to the context of CHWs (Gureje et al. 2019). On the other hand, well designed or culturally adapted interventions may be integrated into the community-health worker’s regular perinatal visits that already existing in some sub-Saharan African settings (Lunsford et al. 2015, Mupara et al. 2023).

### Strength and limitations of the findings

To our best knowledge, this is the first review to identify and report on psycho-social interventions led by CHWs focused on perinatal depression in the sub-Saharan African region. The review is an important contribution both in reporting on the available evidence and indicating direction for future research. The review responded to the unique needs of the sub-Saharan Africa, where evidence related to the work of CHWs across various interventions to prevent and manage perinatal depression is highly needed. The review utilized a systematic and comprehensive search strategy to identify studies conducted in the region. From the meta-analysis, we observed improvements in precision and power to detect changes as compared to individual studies. In addition, the review generated summary of findings table which may aid in making decisions essential to policy and practice related to the role of CHWs in the prevention and management of perinatal depression in sub-Saharan Africa. While the review was based on *a priori* prepared protocol, the protocol for the review was not registered/published. It is also essential to note that the review covered only papers published in the English language. Reports published in languages other than English, such as reports from Francophone countries might have been missed.

Another important area of gap in the existing literature from sub-Saharan Africa is the lack of interventions that use innovative approaches, such telehealth interventions. Furthermore, nearly, two-third of the articles included in this review are from one country (South Africa) and seven of the studies reported on a single intervention but in different settings, populations, and data points. This indicates a significant gap in evidence from other countries of sub-Saharan Africa and underscores the need for additional studies.

## Conclusion

Overall, the existing evidence indicates that the work of CHWs may be integrated both in the prevention and management of perinatal depression with careful contextual considerations.

### Recommendation for practice

Overall, low-quality evidence supports the use of community-health workers to reduce the risk of perinatal depression. This implies additional analysis of the feasibility, applicability and meaningfulness of the interventions to the local context should be considered while adapting the interventions. Moderate-quality evidence indicates that psycho-social therapeutic interventions delivered by CHWs lower depressive symptoms. With intensive training and supervision, CHWs may play active roles in the reduction of depressed mood and depressive symptoms during perinatal period.

### Recommendations for research

Further high-quality randomized trials should be conducted to generate evidence on the effectiveness of interventions conducted by CHWs on perinatal depression. Future studies should use adequate sample size (powered enough to detect differences in dichotomous outcomes).

## Supplementary Material

czaf084_Supplementary_Data

## Data Availability

All the data underlying this article are either directly included in the published article or are provided in the supplementary materials, which are available alongside the article.

## Reflexivity statement

The work produced in this manuscript involved authors from diverse backgrounds. The group is balanced in terms of gender, seniority, professional and geographical origin and experience. Two of the authors are females and three of them are males. The first author is a public health scientist with specialty in evidence-based healthcare with extensive research on perinatal mental health and CHWs. The second author specializes in Social Epidemiology with focus on social justice and health. The third specializes in Integrated Clinical and Community Mental Health and has extensive clinical, community and research experience on mental health. The fourth author is a senior undergraduate Public Health student who had internship experience in sub-Saharan Africa (Zambia) as part of the Brooklyn College’s Tow International Public Health Internship. The last author is a public health expert with focus on social construction of illness, health disparities, and thanatology. Except one author, the remaining authors are senior scientists and experts, two of which are originally from sub-Saharan Africa and have extensive research and service experience on perinatal mental health and CHWs in sub-Saharan Africa.
