# Supplementary material for: How effective are community health workers in managing and preventing perinatal depression in sub-Saharan Africa? A systematic review of quantitative evidence
Source: Health Policy Plan. 2025 Oct 30;41(1):94–116. doi: 10.1093/heapol/czaf084 (PMC12828706; doi:10.1093/heapol/czaf084)
Supplement: czaf084_Supplementary_Data [file czaf084_supplementary_data.zip › Supplementary File 2_Search Strategy clean.docx]

# Supplementary File 2: Search Strategy

**PubMed (last searched on September 16, 2024)**

| S/N | Search | Results |
| --- | --- | --- |
| 1 | "Perinatal Care"[Mesh] OR "Pregnant Women"[Mesh] OR "Prenatal Care"[Mesh] OR “Postnatal Care"[Mesh] OR "Postpartum Period"[Mesh] | 134,129 |
| 2 | Pregnan* [tw] OR Prenatal* [tw] OR antenatal* [tw] OR Perinatal* [tw] OR postnatal* [tw] | 1,334,308 |
| 3 | "Community Health Workers"[Mesh] OR "Community Mental Health Services"[Mesh] OR “Volunteers” [Mesh] | 65048 |
| 4 | ‘Community health workers’ [tw] OR ‘Lay health workers’ [tw] OR “Community health volunteers” [tw] OR ‘Village health workers’ [tw] OR ‘community health aides’ [tw] OR ‘Social Workers’ OR ‘Lay counsel*’ | 12099 |
| 5 | ("Depression"[Mesh] OR "Depressive Disorder"[Mesh] OR "Depressive Disorder, Major"[Mesh] OR "Adjustment Disorders"[Mesh] OR "Psychiatric Status Rating Scales"[Mesh] OR "Affective Disorders, Psychotic"[Mesh] OR "Major Depressive Disorder 1" [Supplementary Concept] OR "Major Depressive Disorder 2" [Supplementary Concept] OR “Patient Health Questionnaire"[Mesh]) OR "Depression, Postpartum"[Mesh] | 331,941 |
| 6 | Depressi* [tw] | 602,524 |
| 7 | 1 OR 2 | 1,360,279 |
| 8 | 3 OR 4 | 70,097 |
| 9 | 5 OR 6 | 656,080 |
| 10 | **7 AND 8 AND 9** | 195 |
| 11 | "Africa South of the Sahara"[Mesh] | 273,253 |
| 12 | "Sub-Saharan" [tw] OR Africa [tw] OR Angola [tw] OR Benin [tw] OR Botswana [tw] OR Burundi [tw] OR “Burkina Faso” [tw] OR “Cabo Verde” OR Cameroon [tw] OR Chad [tw] OR Comoros [tw] OR Congo [tw] OR Côte d'Ivoire OR Ivory Coast OR Djibouti [tw] OR Eritrea [tw] OR “Equatorial Guinea” [tw] OR Eswatini OR Ethiopia [tw] OR Gabon [tw] OR Lesotho [tw] OR Gambia [tw] OR Ghana [tw] OR Guinea [tw] OR Guinea-Bissau [tw] OR Kenya [tw] OR Liberia [tw] OR Madagaskar [tw] OR Mali [tw] OR Mauritania [tw] OR Mozambique [tw] OR Namibia [tw] OR Niger [tw] OR Nigeria [tw] OR Rwanda [tw] OR Sao Tome and Principle [tw] OR Senegal [tw] OR Seychelles OR Sierra Leone [tw] OR Somalia [tw] OR Somali Land [tw] OR “South Africa” OR “South Sudan” [tw] OR Sudan [tw] OR Swaziland OR Tanzania [tw] OR Togo [tw] OR Uganda [tw] OR Zambia [tw] OR Zimbabwe [tw] | 687,312 |
| 13 | **11 OR 12** | 695,384 |
| 14 | **10 AND 13** | 63 |
| 15 | Limit 14 to ENGLISH and Humans | 63 |

**CINAHL (last searched on September 16, 2024)**

| S/N | Search | Results |
| --- | --- | --- |
| 1 | TI (pregnancy OR Pregnant OR Prenatal OR prenatally OR Antenatal OR antenatally OR Postnatal OR postnatally OR postpartum OR perinatally OR Perinatal) OR AB (pregnancy OR Pregnant OR Prenatal OR prenatally OR Antenatal OR antenatally OR Postnatal OR postnatally OR postpartum OR perinatally OR Perinatal) | 239,625 |
| 2 | (MM "Perinatal Period") OR (MM "Perinatal Care") OR (MH "Perinatal Mood and Anxiety Disorders+") OR (MM "Depression, Postpartum") | 11,671 |
| 3 | (MH "Pregnancy Trimesters+") OR (MM "Pregnancy") | 18,430 |
| 4 | (MM "Prenatal Care") OR "antenatal" | 26,926 |
| 5 | (MH "Postnatal Care+") OR (MH "Postnatal Period+") OR (MM "Edinburgh Postnatal Depression Scale") | 24,515 |
| 6 | (MM "Community Health Workers") OR (MH "Community Mental Health Services+") | 15,927 |
| 7 | (MM "Social Workers") OR (MH "Lay Midwives") OR (MH "Lay Midwifery") OR (MM "Volunteer Workers") | 13,773 |
| 8 | TI ( 'lay health worker' OR 'Community health worker' OR 'Social worker' OR 'volunteers' ) OR AB ( 'lay health worker' OR 'Community health worker' OR 'Social worker' OR 'volunteers' OR village health workers) | 65,013 |
| 9 | (MM "Depression") OR TI Depress* OR AB Depress* | 198,123 |
| 10 | S1 OR S2 OR S3 OR S4 OR S5 | 254,652 |
| 11 | S6 OR S7 OR S8 | 85,458 |
| 12 | S9 AND S10 AND S11 | 228 |
| 13 | Language: English; English Language; Exclude MEDLINE records; Human | 82 |
| 14 | Limit to Africa | 0 |

**Database of ProQuest Dissertation and Theses (Last search date: September 13, 2024)**

| Search Query | Results |
| --- | --- |
| Depress* AND (Postpartum OR antenatal OR prenatal OR Perinatal OR pregnancy OR pregnant) AND (Community health worker OR lay health workers OR volunteer OR Social workers OR village health workers) AND English and Africa | 58 |

**SCOPUS (Last search date: September 15, 2024)**

| Search Query | Results |
| --- | --- |
| Depress* AND (Postpartum OR antenatal OR prenatal OR Perinatal OR pregnancy OR pregnant) AND (Community health worker OR lay health workers OR volunteer OR Social workers OR village health workers) limited to Sub Saharan Africa and English | 75 |

**Search results from all Sources**

| Sources | Total articles |
| --- | --- |
| PubMed | 66 |
| CINHAL | 0 |
| SCOPUS | 75 |
| Database of ProQuest Dissertation and Theses | 58 |
| Total records before removing duplicates | 199 |
| Duplicates | 33 |
| Total after removing duplicates | 166 |
