# Supplementary material for: How effective are community health workers in managing and preventing perinatal depression in sub-Saharan Africa? A systematic review of quantitative evidence
Source: Health Policy Plan. 2025 Oct 30;41(1):94–116. doi: 10.1093/heapol/czaf084 (PMC12828706; doi:10.1093/heapol/czaf084)
Supplement: czaf084_Supplementary_Data [file czaf084_supplementary_data.zip › Supplementary File 3 Full text Studies Excluded with Reason Clean.docx]

## **Supplementary File 3: Studies Excluded with Reason**

| Study | Reason for Exclusion |
| --- | --- |
| Christodoulou 2019 | Depression was not used as a dependent variable The authors used depression not as a primary dependent variable, but as a mediating variable to estimate its role as a mediator variable in determining the effect of the home visit intervention on the quality of mother- child interactions. |
| Tomlinson 2018 | Depression was not used as a dependent variable. |
| Tomlinson 2015 | Depression was not a dependent variable. The study sought the moderation effect of depression on child health outcomes |
| Rotheram-Borus 2015 | The study focuses on pathway analysis than comparing the two arms; the effect of the intervention has been presented in Rotheram-Borus 2014 and Tomlinson 2016 |
| Gureje 2019 | The study included non-pregnant study population |
| Oyekuno 2021 | The study included non-pregnant study population; intervention was delivered by non-CHWs |
| Bryant 2017 | The population also included non-perinatal women who experienced GBV |
| Tsai 2014 | No intervention of interest |
| Nakku 2021 | Intervention provided by non-CHWs |
| Spelke 2022 | Intervention provided by non-CHWs |
| Rotherman-Borus 2011 | The report contains only baseline results |
| Lira 2015 | The intervention population included was non-perinatal women (2 years after birth) |
